# Using Digital Tools to Study the Health of Adults Born Preterm at a Large Scale: e-Cohort Pilot Study

**DOI:** 10.2196/39854

**Published:** 2023-05-15

**Authors:** Elsa Lorthe, Carolina Santos, José Pedro Ornelas, Julia Nadine Doetsch, Sandra C S Marques, Raquel Teixeira, Ana Cristina Santos, Carina Rodrigues, Gonçalo Gonçalves, Pedro Ferreira Sousa, João Correia Lopes, Artur Rocha, Henrique Barros

**Affiliations:** 1 EPIUnit - Instituto de Saúde Pública Universidade do Porto Porto Portugal; 2 Laboratório para a Investigação Integrativa e Translacional em Saúde Populacional (ITR) Porto Portugal; 3 Institute for Systems and Computer Engineering, Technology and Science Porto Portugal; 4 NOVA Institute of Communication (ICNOVA) NOVA University of Lisbon Lisboa Portugal; 5 Departamento de Ciências da Saúde Pública e Forenses e Educação Médica Faculdade de Medicina Universidade do Porto Porto Portugal; 6 Department of Informatics Faculty of Engineering University of Porto Porto Portugal

**Keywords:** e-cohort, prematurity, preterm birth, life course epidemiology, participant involvement, citizen science, Research on European Children and Adults born Preterm, RECAP Preterm, premature, preterm, cohort study, epidemiology, observational study, global health, global burden, survey, questionnaire, health outcome, mobile phone

## Abstract

**Background:**

Preterm birth is a global health concern. Its adverse consequences may persist throughout the life course, exerting a potentially heavy burden on families, health systems, and societies. In high-income countries, the first children who benefited from improved care are now adults entering middle age. However, there is a clear gap in the knowledge regarding the long-term outcomes of individuals born preterm.

**Objective:**

This study aimed to assess the feasibility of recruiting and following up an e-cohort of adults born preterm worldwide and provide estimations of participation, characteristics of participants, the acceptability of questions, and the quality of data collected.

**Methods:**

We implemented a prospective, open, observational, and international e-cohort pilot study (Health of Adult People Born Preterm—an e-Cohort Pilot Study [HAPP-e]). Inclusion criteria were being an adult (aged ≥18 years), born preterm (<37 weeks of gestation), having internet access and an email address, and understanding at least 1 of the available languages. A large, multifaceted, and multilingual communication strategy was established. Between December 2019 and June 2021, inclusion and repeated data collection were performed using a secured web platform. We provided descriptive statistics regarding participation in the e-cohort, namely, the number of persons who registered on the platform, signed the consent form, initiated and completed the baseline questionnaire, and initiated and completed the follow-up questionnaire. We also described the main characteristics of the HAPP-e participants and provided an assessment of the quality of the data and the acceptability of sensitive questions.

**Results:**

As of December 31, 2020, a total of 1004 persons had registered on the platform, leading to 527 accounts with a confirmed email and 333 signed consent forms. A total of 333 participants initiated the baseline questionnaire. All participants were invited to follow-up, and 35.7% (119/333) consented to participate, of whom 97.5% (116/119) initiated the follow-up questionnaire. Completion rates were very high both at baseline (296/333, 88.9%) and at follow-up (112/116, 96.6%). This sample of adults born preterm in 34 countries covered a wide range of sociodemographic and health characteristics. The gestational age at birth ranged from 23+6 to 36+6 weeks (median 32, IQR 29-35 weeks). Only 2.1% (7/333) of the participants had previously participated in a cohort of individuals born preterm. Women (252/333, 75.7%) and highly educated participants (235/327, 71.9%) were also overrepresented. Good quality data were collected thanks to validation controls implemented on the web platform. The acceptability of potentially sensitive questions was excellent, as very few participants chose the “I prefer not to say” option when available.

**Conclusions:**

Although we identified room for improvement in specific procedures, this pilot study confirmed the great potential for recruiting a large and diverse sample of adults born preterm worldwide, thereby advancing research on adults born preterm.

## Introduction

### Background

Preterm birth, defined as a birth occurring before 37 weeks of gestation, is a global health concern. It has shown an upward trend since 1990 and accounted in 2014 for 10.6% (uncertainty interval 9.0%-12.0%) of births worldwide, representing almost 15 million babies born preterm every year [1,2]. Large regional and national variations have been reported, ranging from approximately 5% in several European countries to 15% to 20% in many African and Asian countries [1,2].

Preterm birth remains a major cause of mortality during both the neonatal period and childhood [3]. This is particularly true in low- and middle-income countries because of the lack of care and facilities for premature babies [4]. In high-income countries, survival rates have improved over the last 40 years owing to advances in antenatal and neonatal care, reaching 80% to 90% nowadays [5,6]. Among survivors, the frequency of prematurity-related morbidity and health problems is substantial, both in the short term and long term after birth [2,7,8]. Neonatal complications may be transient or become chronic. The effect of preterm birth may thus continue throughout life, exerting a potentially heavy burden on families, health systems, and societies [4].

In high-income countries, the first children who benefited from improved care are now adults entering middle age [9]. With increasing prematurity and survival rates, these “adults born preterm” represent a growing share of the population. A substantial body of research has emerged, focusing on studying the health and well-being of preterm-born babies when they have grown up and become adults [10]. Epidemiological studies, complemented by fundamental research investigating biological mechanisms, have suggested that adults born preterm are likely to experience health problems more often than those born at term. For instance, a higher frequency of cardiovascular and metabolic disorders has been reported, possibly explained by a smaller size at birth and a rapid catch-up in growth [7]. Likewise, a higher frequency of mental health problems, such as anxiety and depression, and reduced social functioning were identified when compared with adults born at term, with multiple and intertwined potential underlying mechanisms, including neurobiological, endocrinological, and psychosocial processes [11]. Furthermore, new life tasks arise in the transition to adulthood (eg, independent living, holding down a job, and creating one’s own family), and evidence of the potential difficulties faced by adults born preterm is sparse. However, if some health risks associated with preterm birth extend across the life span [8,11], most children born very preterm (<32 weeks of gestation) adjust remarkably well during their transition into adulthood [7,12,13].

There is still a clear gap in the knowledge regarding the long-term outcomes of individuals born preterm. Most available data come from cohorts of preterm or high-risk infants initiated in high-income countries between the late 1970s and the early 1990s, with data collection continuing until adult age [9,14-16]. These longitudinal studies, with regular follow-up evaluations over time, are the methodology of reference for assessing the prognosis of high-risk children and the determinants of long-term adverse outcomes. They have already provided major insights into adult outcomes of people born preterm [7,8,12]. However, the number of participants usually has an order of magnitude of a few hundred, which represents a small sample size to study some rare outcomes or analyze subgroups. Furthermore, regular evaluations are time-consuming and money consuming, and attrition over time can bias their results.

The availability of technologies offers new possibilities for research, leading to the development of “e-epidemiology” [17]. The internet opens the door to large-scale epidemiological studies, facilitating participant recruitment, cost-efficient data collection, and dissemination of results [18]. Moreover, the patient’s role in medical research is progressively moving from a subject of study to an actor involved in their own follow-up and a partner for clinicians and researchers [19,20]. To date, this approach has not been applied to individuals born preterm.

### Objectives

Taking advantage of e-epidemiology tools, we implemented a pilot cohort study aiming to (1) assess the feasibility of recruiting and following up an e-cohort of adults born preterm worldwide and (2) provide estimations of participation, characteristics of participants, the acceptability of questions, and the quality of data collected.

## Methods

### Study Design

Health of Adult People Born Preterm—an e-Cohort Pilot Study (HAPP-e) is a prospective, open, observational, international, e-cohort pilot study of adults born preterm. Inclusions in the pilot study started on December 16, 2019. A follow-up evaluation was launched on December 4, 2020. This collaborative project was developed by the Institute of Public Health of the University of Porto (ISPUP) on the epidemiological side and the Institute for Systems and Computer Engineering, Technology and Science on the technical side as part of the Research on European Children and Adults Born Preterm project (RECAP Preterm) [21].

### Participants

The inclusion criteria were as follows: being an adult (aged ≥18 years), born preterm (<37 weeks of gestation), having internet access and an email address, and being able to read and understand at least 1 of the available languages. Here, we reported the data of the participants who registered from December 16, 2019, to December 31, 2020, and who were invited to participate in the follow-up evaluation until June 30, 2021.

### Recruitment

A multifaceted communication strategy was implemented with the aim of disseminating the project’s existence and encouraging the participation of as many and as diverse individuals as possible, with the support of the ISPUP communication office and the European Foundation for the Care of Newborn Infants. A logo and a visual identity were created and used for all communication purposes.

**Figure 1:**
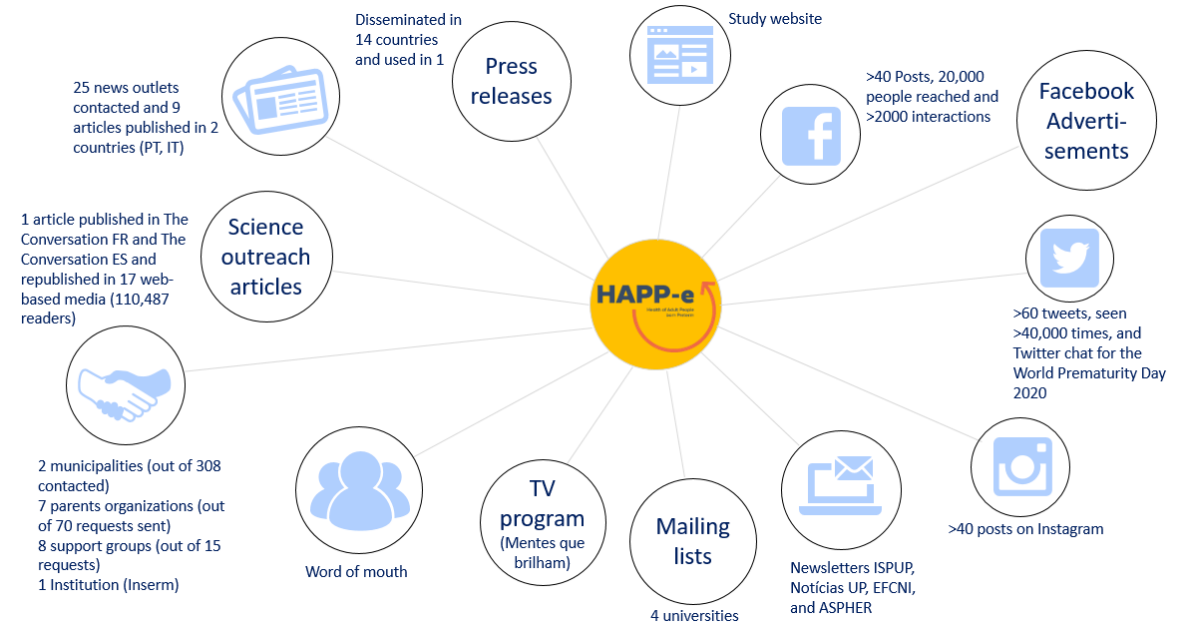
Overview of multifaceted communication strategy. ASPHER: Association of Schools of Public Health in the European Region; EFCNI: European Foundation for the Care of Newborn Infants; ES: España (Spain); FR: France; HAPP-e: Health of Adult People Born Preterm—an e-Cohort Pilot Study; ISPUP: Institute of Public Health of the University of Porto; IT: Italy; PT: Portugal; UP: University of Porto.

The HAPP-e website [22] was the entry point of access for all interested persons and potential participants. It provided relevant information regarding the pilot study’s objectives, design, data processing, and regulations as well as information on preterm birth and consequences until adulthood. To improve accessibility to the broadest international audience possible, all website content, consent forms, and questionnaires were provided in several languages, namely, English, Portuguese, French, German, Spanish (released on July 10, 2020), and Italian (released on September 25, 2020). Visually attractive and based on infographics, the website allowed a nonspecialist audience to access easily understandable information. As shown in Figure 1, we also communicated through repeated and ad hoc actions via social media (mainly Facebook, Instagram, and Twitter, with weekly to biweekly posts since January 2020), traditional media (press releases, press articles, and science outreach articles—with many republications over time in web-based media), television programs, institutional newsletters, and institutional web communications. We developed relationships with the organizations of parents of preterm babies (eg, Prematuridade and SOS prémas) and newly created associations of adults born preterm (eg, Adult Preemie Advocacy Network), who supported us in disseminating the project to their networks. We participated in several web-based events for the World Prematurity Day (November 17, 2020). Social networks were also used as community outreach tools, providing study updates and relevant information and testimonies.

### Inclusion

Inclusion and repeated data collection were performed through a privacy-preserving modular web platform on a server secured by a digital certificate, accessible from the HAPP-e website.

Participation implied a 2-step process. First, a registration module displayed a privacy policy statement and asked for an email address, password, preferred language, and the confirmation of the eligibility criteria. An email was sent automatically with a link to confirm the registration. On logging in, the participants had to comply with a 2-factor authentication implemented by the platform by providing the selected credentials and accessing a link on their email that granted them access to the platform. Second, inside the platform, the first module displayed a detailed information form. After reading this form, adults born preterm were invited to confirm eligibility criteria and electronically sign an informed consent form to participate in the e-cohort. Finally, the participants accessed a second module and completed the baseline questionnaire, which was divided into several subsections to improve their experience. Any step uncompleted after 1 day triggered a reminder sent via email once a week for up to 3 weeks. Accounts were deleted if the email address was not confirmed after 15 days.

All participants who had been in the cohort for >6 months were invited to the follow-up evaluation via email. Once connected to their personal area using a 2-factor authentication, they electronically signed a new informed consent form before completing the follow-up questionnaire. In the case of nonresponse, email reminders were sent once a week for up to 3 weeks.

### Data Collection

The baseline questionnaire had a total of 6 sections, with 302 questions (median completion time 45, IQR 33-65 minutes); however, most respondents answered fewer questions, as the questionnaire allowed them to skip sections. The following topics were covered: circumstances and conditions of birth and past health conditions, sociodemographic data, education and employment, health data and biometrics, quality of life, mental health, and lifestyle factors.

The follow-up questionnaire had 8 sections, with 138 questions (median completion time 22, IQR 17-32 minutes), which focused on current living conditions and changes since the baseline questionnaire, relationship with parents, stress, current health (including a few questions about the COVID-19 pandemic), health care consumption, mental health, quality of life, and the perceived impact of preterm birth on certain aspects of life. The participants were also invited to express themselves in a few free-text sections.

Some information was collected only once at baseline, namely, regarding domains with no evolution (circumstances of birth and past health condition), whereas other information was also collected at follow-up (for instance, current health or quality of life), to assess evolutions from a longitudinal perspective.

Validated scales and open-access instruments were used as often as possible (Table 1), including those recommended by the Common Core Assessments in follow-up studies of adults born preterm [9]. Real-time automatic validation controls were implemented to improve the quality of data. Validation included checking valid ranges and data formats, signaling missing data, or validating answers based on predefined rules.

All participants received personalized feedback on how they compared themselves with other participants on several dimensions assessed in the inclusion questionnaire. Participants were invited to contribute to the definition of topics of interest for the follow-up evaluation through a survey on Twitter and exchanges with members of the Adult Preemie Advocacy Network. Following this survey, we included questions on mental health, stress, and relationship with parents as well as open questions on the perceived impact of preterm birth on everyday life.

**Table 1:** Main measures and instruments used in the Health of Adult People Born Preterm—an e-Cohort Pilot Study.

Measurement category		Dimensions measured and instruments used	Baseline questionnaire	Follow-up questionnaire
**Sociodemographic**				
	Core social variables	Year and country of birth, gender, mother tongue, nationality, and ethnicity; European Health Interview Survey (wave 3, 2018 edition)	✓^a^	
	Education	United Nations Educational, Scientific and Cultural Organization (UNESCO) International Standard Classification of Education (ISCED), 2011	✓	✓ (change from baseline)
	Employment	International Labour Organization International Standard Classification of Occupations 2008 (ISCO-08); European Health Interview Survey (wave 3, 2018 edition)	✓	✓ (change from baseline)
	Current living conditions	Country of residence, marital status, household structure, socioeconomic class, and subjective personal finance; European Health Interview Survey (wave 3, 2018 edition)	✓	✓ (change from baseline)
	Parents	Country of birth, education, and employment	✓	
	Relationship with parents	Parental Bounding Instrument		✓
	Social support	Multidimensional Scale of Perceived Social Support	✓	
**Health**				
	Circumstances of birth	Gestational age, birth weight, type of pregnancy, cause of preterm birth, and mode of delivery	✓	
	Past health condition	Neonatal morbidity, prematurity-related morbidity during childhood, and past mental health problems	✓	
	Biometrics	Height and weight; European Health Interview Survey (wave 3, 2018 edition)	✓	
	Current general health	Self-perceived general health, long-standing health problems, limitations in activities, diseases in the past 12 months, pain, medications, and functional limitations; Minimum European Health Module; European Health Interview Survey (wave 3, 2018 edition)	✓	✓
	Use of medications	European Health Interview Survey (wave 3, 2018 edition)	✓	✓
	Oral health	European Health Interview Survey (wave 3, 2018 edition)	✓	
	Sexual and reproductive health	Sexual orientation, infertility, and children; for women only: pregnancy history (including giving birth to a preterm baby)	✓	
	Mental health	DSM-5^b^ Self-Rated Level 1 Cross-Cutting Symptom Measure—Adult	✓	✓
	Health care consumption	Follow-up program after preterm birth, hospitalizations, medical consultations, and unmet needs for health care; European Health Interview Survey (wave 3, 2018 edition)		✓
	Vulnerability to illness	Perceived Vulnerability to Illness Scale		✓
	COVID-19	Symptoms, diagnostic tests, serological tests, and quarantine		✓
**Psychosocial**				
	Personality	Big Five Inventory-10	✓	
	Quality of life	Assessment of quality of life - 8D	✓	✓
	Stress	Perceived Stress Scale		✓
	Resilience	Brief Resilience Scale (partial)	✓	
	Well-being	Short Warwick–Edinburgh Mental Well-being Scale	✓	✓
	Satisfaction with life	Satisfaction With Life Scale	✓	
	Bullying	Bullying self-report scale by Wolke et al [8]	✓	
**Lifestyle**				
	Smoking	European Health Interview Survey (wave 3, 2018 edition)	✓	
	Alcohol consumption	WHO^c^ Alcohol Use Disorders Identification Test	✓	
	Drugs use	WHO Alcohol, Smoking and Substance Involvement Screening Test (1 item)	✓	
	Physical activity	International Physical Activity Questionnaire—short-form	✓	
	Sleep	Pittsburgh Sleep Quality Index	✓	

^a^Item evaluated in that questionnaire.

^b^DSM-5: Diagnostic and Statistical Manual of Mental Disorders, Fifth Edition.

^c^WHO: World Health Organization.

### Study Outcome Measures

We reported participation in the e-cohort, namely, the number of persons who (1) registered on the platform, (2) signed the baseline consent form, (3) initiated and completed the baseline questionnaire, (4) signed the follow-up consent form, and (5) initiated and completed the follow-up questionnaire. To assess the geographical catchment area associated with the study, participants’ birth countries were mapped. We also described the main characteristics of the HAPP-e participants and provided an assessment of the quality of data (proportion of missing data) and the acceptability of sensitive questions (assessed by the proportion of participants who answered “I prefer not to say”).

### Statistical Analysis

Descriptive analyses of the selected questions collected in the pilot study were conducted to inform the study protocol for a subsequent study. Participant characteristics were described as frequencies and percentages, means and SDs, or medians and IQRs. We compared sociodemographic characteristics by mode of recruitment using chi-square or Fisher exact tests, as appropriate. The validated scales were scored and interpreted according to the recommendations of the authors. We restricted our analyses to the available data. Statistical significance was set at a 2-tailed value of *P*<.05. Data were analyzed using Stata/SE 16.0 (StataCorp LLC).

### Ethics Approval

This pilot study was reviewed and approved by the ISPUP Research Ethics Committee on July 25, 2019 (CE19124). All participants were informed about the study and the confidentiality of their data and electronically signed a consent form before participating in the study at baseline and at follow-up.

## Results

### Participation

**Figure 2:**
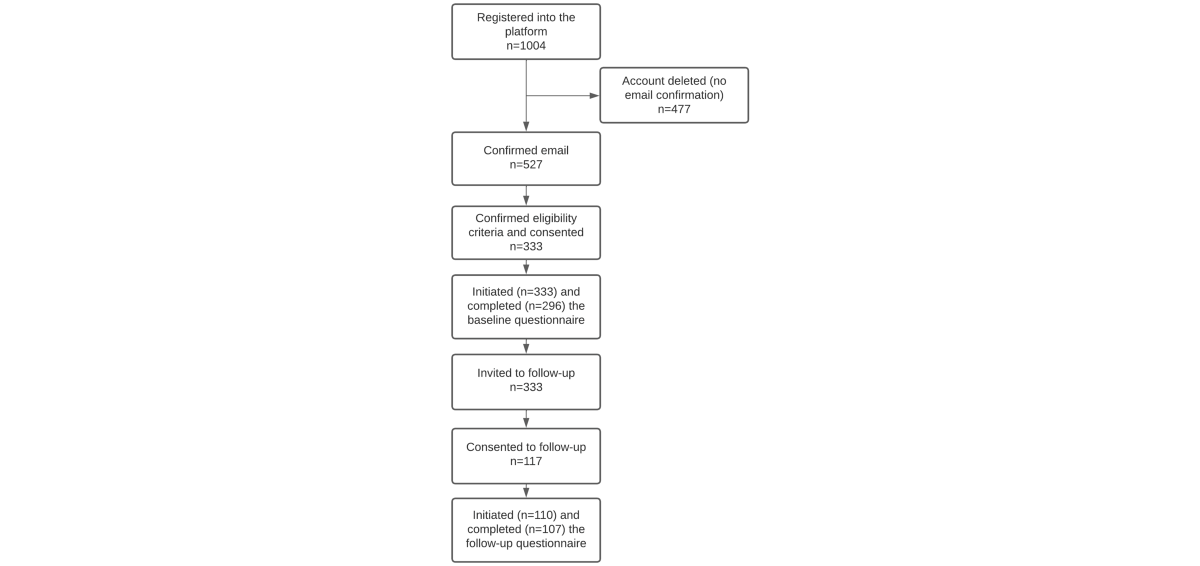
Flowchart of participants enrolled in the Health of Adult People Born Preterm—an e-Cohort Pilot Study.

As of December 31, 2020, a total of 1004 persons had registered on the platform, and 527 accounts were considered valid (ie, with a confirmed email), among which 333 participants signed the consent form (Figure 2). All participants initiated the baseline questionnaire, and 88.9% (296/333) of the participants completed it. These 333 persons were invited to follow-up, and 35.7% (119/333) of the participants consented to participate, of whom 97.5% (116/119) initiated the follow-up questionnaire and 96.6% (112/116) completed it.

The number of baseline questionnaires administered per month from December 2019 to December 2020 was as follows: 4, 33, 120, 27, 8, 10, 10, 13, 11, 21, 8, 33, and 35.

### Description of Participants at Baseline

#### Sociodemographic Characteristics

**Figure 3:**
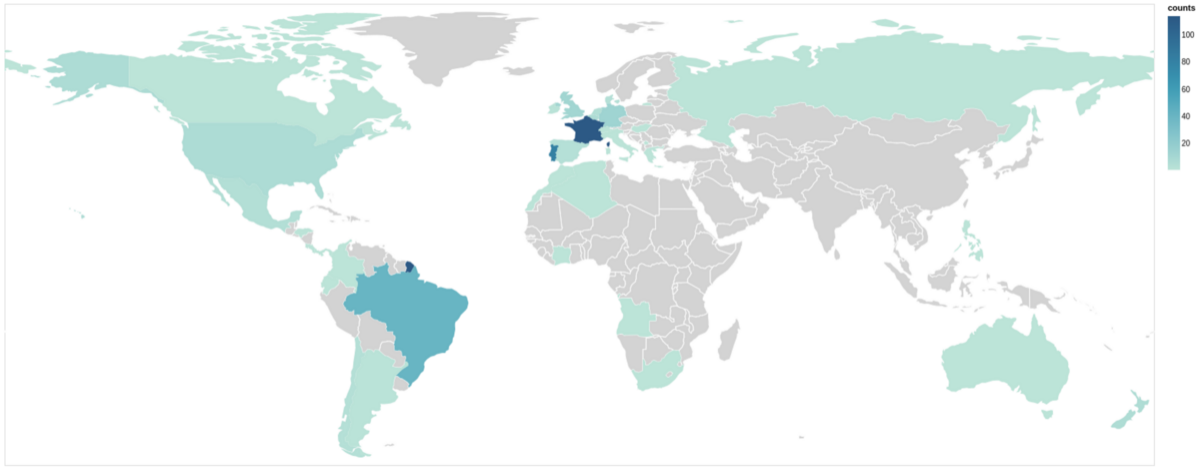
Country of birth of participants enrolled in the Health of Adult People Born Preterm—an e-Cohort Pilot Study. The colors correspond to the number of participants per country.

The participants were born in 34 countries from 6 continents (Figure 3). The most represented countries were France (114/333, 34.2%), Portugal (83/333, 24.9%), Brazil (42/333, 12.6%), Germany (14/333, 4.2%), and the United Kingdom (13/333, 3.9%). The age range was 18-85 years, with a median age at baseline of 30 (IQR 24-40) years. Most participants were women (252/333, 75.7%) and highly educated; 30.3% (99/327) had a bachelor’s degree, 31.2% (102/327) had a master’s degree, and 10.4% (34/327) had a doctoral degree (Table 2).

**Table 2:** Sociodemographic characteristics of participants enrolled in the Health of Adult People Born Preterm—an e-Cohort Pilot Study (n=333).

Characteristic			Values
**Gender (n=333), n (%)**			
	Man	79 (23.7)	
	Woman	252 (75.7)	
	Other	2 (0.6)	
**Year of birth (n=333), n (%)**			
	1935-1939	1 (0.3)	
	1940-1949	3 (0.9)	
	1950-1959	9 (2.7)	
	1960-1969	29 (8.7)	
	1970-1979	41 (12.3)	
	1980-1989	80 (24)	
	1990-1999	141 (42.4)	
	2000-2002	29 (8.7)	
**Education (n=327), n (%)**			
	None	1 (0.3)	
	Primary education	0 (0)	
	Lower secondary education	5 (1.5)	
	Upper secondary education	53 (16.2)	
	Postsecondary nontertiary education	8 (2.5)	
	Short-cycle tertiary education	24 (7.3)	
	Bachelor’s degree	99 (30.3)	
	Master’s degree	102 (31.2)	
	Doctorate	34 (10.4)	
	Not classified	1 (0.3)	
**Current main activity status (n=327), n (%)**			
	Employed	182 (55.7)	
	Unemployed	23 (7)	
	Retired	12 (3.7)	
	Unable to work owing to health problems	5 (1.5)	
	Student	88 (26.9)	
	Fulfilling domestic tasks	5 (1.5)	
	Other	12 (3.7)	
	I prefer not to say	0 (0)	
**If employed, main occupation (n=175), n (%)**			
	Manager	5 (2.9)	
	Professional	115 (65.7)	
	Technician and associate professionals	12 (6.8)	
	Clerical support worker	15 (8.6)	
	Services and sales worker	9 (5.1)	
	Skilled agricultural, forestry, and fishery worker	1 (0.6)	
	Craft and related trades worker	2 (1.1)	
	Plant and machine operator and assembler	1 (0.6)	
	Armed-forces occupation	1 (0.6)	
	Other	14 (8)	
**Social support (Multidimensional Scale of Perceived Social Support; n=327), mean (SD)**			
	Significant other subscale	5.86 (1.31)	
	Family subscale	5.40 (1.50)	
	Friends subscale	5.50 (1.32)	
	Total scale	5.71 (1.05)	

Participants were informed about the study through different channels: social media or websites (111/333, 33.3%), traditional media (52/333, 15.6%), associations (36/333, 10.8%), word of mouth or health care professionals (58/333, 17.4%), or other (76/333, 22.8%). We observed no differences in gender or education by mode of recruitment (Table 3). However, older adults were more likely to be recruited through traditional media, whereas younger and non-European participants joined mainly through social media communication.

**Table 3:** Sociodemographic characteristics by mode of recruitment (n=333).

Characteristic		Social media^a^ (n=111), n (%)	Traditional media^b^ (n=52), n (%)	Association^c^ (n=36), n (%)	Contact^d^ (n=58), n (%)	Other (n=76), n (%)	*P* value	
**Gender (n=333)**								.29
	Man	21 (26.6)	11 (13.9)	10 (12.7)	17 (21.5)	20 (25.3)		
	Woman	90 (35.7)	41 (16.3)	25 (9.9)	40 (15.9)	56 (22.2)		
	Other	0 (0)	0 (0)	1 (50)	1 (50)	0 (0)		
**Year of birth (n=333)**								.005
	1935-1939	0 (0)	0 (0)	0 (0)	0 (0)	1 (100)		
	1940-1949	2 (66.7)	0 (0)	0 (0)	0 (0)	1 (33.3)		
	1950-1959	0 (0)	3 (33.3)	1 (11.1)	2 (22.2)	3 (33.4)		
	1960-1969	4 (13.8)	12 (41.4)	5 (17.2)	5 (17.2)	3 (10.4)		
	1970-1979	13 (31.7)	7 (17)	4 (9.8)	4 (9.8)	13 (31.7)		
	1980-1989	38 (47.4)	13 (16.3)	4 (5)	12 (15)	13 (16.3)		
	1990-1999	49 (34.8)	14 (9.9)	18 (12.8)	27 (19.1)	33 (23.4)		
	2000-2002	5 (17.2)	3 (10.4)	4 (13.8)	8 (27.6)	9 (31)		
**Education (n=326)**								.25
	Low (up to 12th grade)	13 (22)	8 (13.6)	9 (15.3)	12 (20.3)	17 (28.8)		
	High (postsecondary or tertiary education)	95 (35.6)	42 (15.7)	27 (10.1)	45 (16.9)	58 (21.7)		
**Continent of residence (n=326)**								<.001
	Europe	59 (23.1)	46 (18)	34 (13.3)	45 (17.7)	71 (27.8)		
	Africa	2 (66.7)	1 (33.3)	0 (0)	0 (0)	0 (0)		
	North America	4 (57.1)	0 (0)	0 (0)	2 (28.6)	1 (14.3)		
	South America	34 (66.7)	3 (5.9)	2 (3.9)	9 (17.6)	3 (5.9)		
	Oceania	8 (88.9)	0 (0)	0 (0)	1 (11.1)	0 (0)		
	Asia	1 (100)	0 (0)	0 (0)	0 (0)	0 (0)		

^a^Social media: Facebook, Twitter, Instagram, Pinterest, and study website.

^b^Traditional media: press, radio, and television.

^c^Association: institutional newsletters and web communications and prematurity-related associations of patients.

^d^Contact: word of mouth and health care professionals.

#### History of Preterm Birth

Only 2.1% (7/333) of the participants had previously participated in a cohort of individuals born preterm. Of note, 70.3% (234/333) of the participants precisely knew their gestational age at birth (in weeks and days), which ranged from 23+6 to 36+6 weeks (median 32, IQR 29-35 weeks). Birth weight ranged from 453 to 3750 g (median 1670, IQR 1100-2182 g). Gestational age and birth weight were unknown in 6.3% (21/333) and 9% (30/333) of the participants, respectively.

#### Past and Current Health

A total of 35.7% (119/333) of the participants declared that they had received a diagnosis of at least 1 morbidity during the neonatal period (Table 4). The most frequently reported neonatal morbidities were respiratory (61/274, 22.3%), infectious (33/274, 12%), and digestive (17/274, 6.2%) complications. Prematurity-related morbidity in childhood was reported by 19.2% (64/333) of participants, particularly cerebral palsy (9/288, 3.1%), hearing impairment (12/288, 4.2%), visual impairment (10/288, 3.5%), and other conditions (41/288, 14.2%). Current general health and oral health were perceived as good or very good by 81.1% (258/318) and 70.1% (223/318) of the participants, respectively. Long-standing health problems were mentioned by 47.2% (150/318) of the participants, including functional limitation in 22.1% (60/318) of the participants and severe functional limitation in 3.1% (10/318) of the participants.

**Table 4:** Past and current health status assessed at baseline (n=333).

Characteristic					Values, n (%)
**Past health**					
	**Neonatal morbidity (n=333)**				
		No	156 (46.9)		
		Yes	119 (35.7)		
		I do not know	57 (17.1)		
		I prefer not to say	1 (0.3)		
	**Type of neonatal morbidity (among participants who answered “Yes” or “No” to the previous question; n=274)**				
		Intraventricular hemorrhage	9 (3.3)		
		Other brain lesion	5 (1.8)		
		Respiratory complication	61 (22.3)		
		Infection	33 (12)		
		Digestive complication	17 (6.2)		
		Visual complication	10 (3.7)		
		Other complication	41 (15)		
	**Prematurity-related morbidity during childhood (n=333)**				
		No	224 (67.3)		
		Yes	64 (19.2)		
		I do not know	44 (13.2)		
		I prefer not to say	1 (0.3)		
	**Type of morbidity during childhood (among participants who answered “Yes” or “No” to the previous question; n=288)**				
		Cerebral palsy	9 (3.1)		
		Visual impairment	10 (3.5)		
		Hearing impairment	12 (4.2)		
		Other	41 (14.2)		
**Current health**					
	**Self-perceived general health (n=318)**				
		Very good	85 (26.7)		
		Good	173 (54.4)		
		Fair	51 (16.1)		
		Bad	9 (2.8)		
		Very bad	0 (0)		
	Long-standing health problem (n=318)			150 (47.2)	
	**Limitation in activities because of health problems (n=318)**				
		No limitation at all	238 (74.8)		
		Limitation but not severe	60 (22.1)		
		Severe limitation	10 (3.1)		
	**Self-perceived general oral health (n=318)**				
		Very good	93 (29.2)		
		Good	130 (40.9)		
		Fair	68 (21.4)		
		Bad	21 (6.6)		
		Very bad	6 (1.9)		

#### Mental Health

Past mental health problems and current long-standing mental health problems were acknowledged by 34.8% (116/333) and 28.7% (89/333) of participants, respectively (Table 5). According to the Diagnostic and Statistical Manual of Mental Disorders, Fifth Edition (DSM-5), Self-Rated Level 1 Cross-Cutting Symptom Measure, which assesses 13 psychiatric domains during the past 2 weeks, the most frequent mental health issues were anxiety (210/310, 67.7%) and depression (156/310, 50.3%).

**Table 5:** Mental health, quality of life, and personality assessed at baseline (n=333).

Characteristic				Values
**Any mental health problem in the past (n=333), n (%)**				
	No		199 (59.8)	
	Yes		116 (34.8)	
	I don’t know		18 (5.4)	
**Current long-standing mental impairments, illness, or disability (n=310), n (%)**				
	No		203 (65.5)	
	Yes		89 (28.7)	
	I don’t know		18 (5.8)	
	I prefer not to say		0 (0)	
**Mental health over the last 2 weeks (DSM-5^a^; n=310), n (%)**				
	Depression—mild or greater		156 (50.3)	
	Anger—mild or greater		133 (42.9)	
	Mania—mild or greater		138 (44.5)	
	Anxiety—mild or greater		210 (67.7)	
	Somatic symptoms—mild or greater		140 (45.2)	
	Suicidal ideation—slight or greater		73 (23.6)	
	Psychosis—slight or greater		32 (10.3)	
	Sleep problems—mild or greater		130 (41.9)	
	Memory issues—mild or greater		53 (17.1)	
	Repetitive thoughts and behaviors—mild or greater		77 (24.8)	
	Dissociation—mild or greater		57 (18.4)	
	Personality functioning issues—mild or greater		128 (41.3)	
	Substance use—slight or greater		107 (34.5)	
**Quality of life (Assessment of Quality of Life-8D; n=305),** **standardized score (0-100), mean (SD)**				
	Independent living		90.6 (11.0)	
	Pain		82.3 (21.4)	
	Senses		83.4 (11.5)	
	Physical superdimension score		86.3 (10.2)	
	Mental health		64.5 (16.8)	
	Happiness		62.7 (17.3)	
	Coping		64.2 (18.8)	
	Relationships		74.3 (17.0)	
	Self-worth		64.4 (22.6)	
	Psychosocial superdimension score		66.8 (16.0)	
	Quality of life total score		72.5 (13.3)	
**Personality (Big Five Inventory-10; n=310),** **mean (SD)**				
	Extraversion		2.75 (1.13)	
	Agreeableness		3.43 (0.85)	
	Conscientiousness		3.66 (0.96)	
	Neuroticism		3.28 (1.03)	
	Openness to experience		3.71 (0.94)	

^a^DSM-5: Diagnostic and Statistical Manual of Mental Disorders, Fifth Edition.

#### Quality of Life

According to the Assessment of Quality of Life-8D, the standardized additive mean scores were 86.3 (SD 10.2) for the physical superdimension and 66.8 (SD 16.0) for the psychosocial superdimension (Table 5).

### Follow-up Evaluation

#### Current Health

During the follow-up evaluation, the current general health was perceived as very good by 26.1% (30/115), good by 51.3% (59/115), fair by 19.1% (22/115), bad by 1.7% (2/115), and very bad by 1.7% (2/115) of the participants. Long-standing health problems were mentioned by 47% (54/115) of the patients, including functional limitation (31/115, 27%) and severe functional limitation (6/115, 5.2%). Overall, 34.8% (40/115) of the respondents reported having had any symptoms suggestive of COVID-19 since January 2020, whereas 6.1% (7/115) had a positive reverse transcription polymerase chain reaction or antigen test, and 3.5% (4/115) had a positive serological test.

#### Mental Health

Current long-standing mental health problems were acknowledged by 23% (26/113) of the participants. The DSM-5 Self-Rated Level 1 Cross-Cutting Symptom Measure was repeated in the follow-up questionnaire, displaying stable results. The most frequent mental health issues were anxiety (77/113, 68.1%) and depression (62/113, 54.9%).

#### Follow-up Program

Overall, 14.9% (17/114) of the respondents participated in a follow-up program for preterm babies when they were children, and 5.3% (6/114) of the respondents participated when they were adolescents. At adult age, 23.7% (27/114) of the participants were asked by their family physician if they were born preterm, whereas 48.3% (55/114) of the participants mentioned their history of preterm birth to their general practitioner. Only 5.3% (6/114) of the participants received specific care or medical examination because they were born preterm.

### Acceptability of Questions and Quality of Data

Owing to the controls implemented in the web platform (skip logics, data entry formatting, intermittent saving, option to resume questionnaire filling, and feedback messages), the completed modules had no missing data. Very few participants chose the “I prefer not to say” option when available (usually <0.5% per question), even for potentially sensitive questions such as ethnicity (0/333, 0%), marital status (1/333, 0.3%), receipt of social benefits (2/327, 0.6%), financial hardship (4/327, 1.2%), current mental health (0/310, 0%), or sexual orientation (9/318, 2.8%).

## Discussion

### Principal Findings

The HAPP-e pilot study was conducted to assess the feasibility of the methods and procedures for recruiting and following up an e-cohort of preterm adults. Over 1 year, >1000 persons were registered on the platform, and 333 consented to participate. Of those who participated in the baseline assessment, 35.7% (119/333) also consented to participate in the follow-up evaluation. The completion rate among those who started to answer the questionnaires was very high in both evaluations, and good-quality data were collected thanks to controls implemented in the web platform. The acceptability of the potentially sensitive questions was high. Establishing such an e-cohort at the international level is therefore feasible, despite some scientific and technical challenges related to the recruitment and retention of participants.

### Comparison With Prior Work

This pilot study demonstrated that a multifaceted communication strategy is crucial for recruiting a sample of adults born preterm, covering a wide range of sociodemographic and health characteristics. The complementarity of the internet and noninternet recruitment methods made it possible to achieve greater diversity in the ages and countries represented among the participants, which is a clear asset when making comparisons over time and geographical areas. This finding is in line with previous studies focusing on other research populations such as pregnant patients or adults during the COVID-19 pandemic [23-25].

A major challenge is that preterm adults are a hard-to-reach group. Indeed, many people do not know their gestational age at birth. Moreover, because being born preterm is not considered a chronic condition yet, most adults born preterm are unaware of the potential long-term consequences and the need for research in this area [26]. This is probably an explanation for the existence of very few organizations of patients, contrary to the associations of parents of preterm babies, which exist in many countries and are federated at an international level. In addition, the vast majority of family doctors do not know about the consequences of preterm birth throughout the life course and, therefore, do not ask about gestational age at birth when taking a medical history [27,28], which was confirmed by our findings.

Participation in this pilot study was patterned by gender and socioeconomic position, with a large overrepresentation of women and highly educated participants. These factors are commonly associated with the decision to participate in research and to maintain participation over time both in longitudinal cohorts of individuals born preterm [29] and in e-cohorts on other topics [18,30]. Although electronic questionnaires are convenient, they require email address availability, internet access, and digital literacy, which are highly dependent on ethnicity and socioeconomic position [31]. Country also had great influence on participation. The underrepresentation of adults who were born preterm in low- and middle-income countries was likely related to the languages available on the platform and the national networks of the investigators, which supported communication and dissemination. Among participants, completion rates were similar or higher than those in other studies [23-25], but follow-up rates were comparable or lower than those in traditional cohorts [32-35]. Comparisons are difficult because attrition is understudied and underreported, including in e-cohorts [33].

Flexible digital procedures have the potential to lower costs by reducing the amount of time study staff members devote to contacting the participants. They may also improve usability and participant satisfaction by offering a convenient method for collecting information. However, disseminating such a study at an international level, solving arising technical problems, dealing with queries from participants, managing data collection instruments, and so on, require a lot of skills, time, financial, and human resources [18], which should be considered in resource-limited environments. Moreover, technical challenges should not be underestimated. For instance, some email providers sent automatic emails (registration confirmation and authentication) to the spam folder, preventing interested persons from completing the process. Along with noneligibility, this may explain the gap between the number of registrations and actual enrollment [24]. Moreover, the absence of personal contact with the study team may imply lower involvement over time, as suggested by this study.

In the absence of any previous cohort of adults born preterm at an international level and because of the aforementioned self-selection biases, the representativeness of our sample is difficult to assess. Most of the characteristics of our volunteers are not directly comparable with existing data on adults born preterm because these data are almost exclusively issued from Scandinavian registries. Unfortunately, to our knowledge, there is no international census or representative sample that allows us to compare and weigh our data. Although we did not include a control sample of term-born adults, the results of our study are in general accordance with the findings published in the medical literature. For instance, compared with term-born controls, preterm populations are at an increased risk of mental health disorders, particularly depressive and anxiety disorders, and are less likely to be extroverted but report equally good social support [8,10]. These data could contribute to advancing knowledge on factors associated with adverse or positive outcomes among adults born preterm, comparing outcomes by gestational age or neonatal complications, identifying trajectories of risk or resilience, and shedding light on late-life outcomes and the aging process [9].

### Strengths and Limitations

This pilot study had several strengths. We carefully respected ethics and security rules, for instance, using transparent, understandable, and detailed web-based consent and secured data infrastructure. We were able to recruit a diverse cohort covering a wide range of gestational ages at birth, year, and country of birth, thanks to a multifaceted communication strategy. Over a year, we recruited >300 participants, which is the same order of magnitude as follow-ups at adult age of existing longitudinal cohorts of individuals born preterm in the 1980s, such as the Bavarian Longitudinal Study [36] or the Project On Preterm and Small-for-Gestational-Age Infants [34,37]. The completion rates were high despite the long baseline questionnaire [38]. Through their participation and positive feedback, the participants demonstrated great interest in better understanding the impact of being born preterm in their daily lives. There is a clear potential in building a community of adults born preterm willing to be actors of medical research and develop a partnership with researchers, thus laying the foundation for a successful coconstruction in a future study [20].

This pilot study also has some limitations to which a further study should pay attention. All study materials were translated into 6 languages; however, this number should be increased to minimize language access issues as much as possible. Technical limitations had a significant impact on registration and dropout rates. For instance, the platform was not designed to be accessed using smartphones, which is the main device used to access the internet in many countries and by young adults. We also noticed a clear drop in inclusion from the beginning of the COVID-19 pandemic, perhaps because attention was mostly on this new disease, new concerns arising from the successive lockdowns, worries about personal and societal situations, and innumerable solicitations for web-based surveys about COVID-19. Although traditional cohorts frequently use a variety of modes of contact and reminders to increase participation in follow-up evaluations [18,39], we were limited because we only had email addresses and could not reasonably consider reaching participants by any other means. This certainly contributed to a rather low follow-up response rate, which could hamper the establishment of a long-term cohort study (ideally over the life course), which is highly relevant in this population. Finally, potential external validity limitations should be considered when interpreting the data presented in this paper.

### Implications for Future Research

On the basis of our experience and the literature, we identified key aspects that should be considered when implementing future studies with a similar design. The platform and questionnaires must be accessible from several types of devices (computer, tablet, and smartphone) and browsers. Adequate technical procedures are required to prevent emails from being considered as spam. The research team should be multicultural and multilingual; include local contact points in several countries; and be reachable via email, phone, and chat services such as WhatsApp. This would support participants with any technical procedures and problems they may encounter, answer queries, update contact details, create personal connections, tailor dissemination strategies, and allow a variety of reminders to be sent (emails, text messages, and phone calls) [40]. Other strategies should be considered to keep participants motivated and engaged in follow-up assessments: using short and personalized e-questionnaires, including a statement that others have responded to and an image in the invitation email, giving a deadline, and providing personalized and understandable feedback [41]. A large-scale multifaceted communication strategy based on both web-based and offline methods is highly valuable. Community managers could also help create engagement via groups on social media by providing study updates, relevant news, and findings on a regular basis and organizing community outreach events (such as webinars). Depending on scientific objectives, a comparison group born at term could be recruited, with additional challenges [9]. Finally, although the flexibility, scalability, and breadth of e-cohorts are undeniable, adequate human and technical resources are required to successfully implement and maintain a large e-cohort over time, all of which are costly [18].

### Conclusions

This pilot study demonstrated the feasibility of recruiting and following up an e-cohort of adults born preterm and provided relevant insights into the specific challenges that will be addressed in a subsequent main study, provided adequate funding is secured. This approach has inherent advantages and limitations and should be considered complementary to traditional cohorts.

## Abbreviations

DSM-5: Diagnostic and Statistical Manual of Mental Disorders, Fifth Edition
HAPP-e: Health of Adult People Born Preterm—an e-Cohort Pilot Study
ISPUP: Institute of Public Health of the University of Porto
RECAP Preterm: Research on European Children and Adults Born Preterm
